# Luteolin 7-Glucuronide in *Artemisia rupestris* L. Extract Attenuates Pulmonary Fibrosis by Inhibiting Fibroblast Activation and FMT via Targeting of TGF-β1

**DOI:** 10.3390/antiox14050533

**Published:** 2025-04-29

**Authors:** Lingfeng Peng, Yimeng Fan, Luyao Wang, Chao Han, Zhihui Hao

**Affiliations:** 1 Chinese Veterinary Medicine Innovation Center, College of Veterinary Medicine, China Agricultural University, Beijing 100193, China; a3524513@163.com (L.P.); 18235970436@163.com (Y.F.); wlyao09@163.com (L.W.); 18730285181@163.com (C.H.); 2 Key Biology Laboratory of Chinese Veterinary Medicine, Ministry of Agriculture and Rural Affairs, Beijing 100193, China; 3 National Center of Technology Innovation for Medicinal Function of Food, National Food and Strategic Reserves Administration, Beijing 100193, China

**Keywords:** *Artemisia rupestris* L., luteolin 7-glucuronide, pulmonary fibrosis, TGF-β1 signaling pathway

## Abstract

Pulmonary fibrosis (PF) is a chronic pulmonary disease characterized by excessive extracellular matrix (ECM) deposition, with cigarette smoking being a major risk factor and no effective treatment at present. Transforming growth factor beta 1 (TGF-β1) plays a key role in PF and regulating oxidative stress. This study investigated the effects and mechanisms of *Artemisia rupestris* L. ethanol extract (ER) on cigarette smoke (CS)-induced PF. We used pull-down and LC–MS analyses to screen and identify compounds that bind to TGF-β1 in ER. We demonstrated that ER inhibits CS-induced PF, lung inflammation, and oxidative stress, thereby improving pulmonary structural injury. The ER inhibits fibroblast activation and fibroblast-to-myofibroblast transition (FMT), reducing collagen deposition for the treatment of PF. We identified the active ingredient in ER that binds to TGF-β1, namely, Luteolin 7-glucuronide (LG). LG inhibits the TGF-β1 signaling pathway through targeted binding to TGF-β1, downregulates the expression of downstream proteins (including collagen I, α-SMA, MMP-2, and MMP-9), and inhibits *fibronectin* expression. It also inhibits fibroblast activation and FMT, enhances *E-cadherin* expression to promote fibroblast adhesion, and suppresses collagen deposition, alleviating PF. Based on these findings, we propose that LG might be a promising therapeutic drug candidate for treating PF.

## 1. Background

Pulmonary fibrosis (PF) is a progressive disorder characterized by alveolar architecture destruction and interstitial fibrosis, ultimately leading to respiratory failure and death. No definitive cure is currently available for PF. Cigarette smoking-induced pulmonary inflammation and oxidative stress leading to structural lung damage form the basis of PF development [1]. Two FDA-approved drugs—nintedanib and pirfenidone—which inhibit fibroblast proliferation and collagen production, are available for the treatment of PF; however, they only slow the disease progression and cannot cure it. Moreover, severe side effects are observed in patients treated with nintedanib and pirfenidone [2,3]. Therefore, there is an urgent need to develop new, more effective drugs for PF.

TGF-β1, a key pro-fibrotic factor, is exacerbated by pulmonary inflammation and oxidative stress, leading to PF [4]. The downstream signaling pathway, TGF-β1-driven PF, primarily involves Smad signaling (Smad2 and Smad3) and non-Smad signaling (MAPK and ERK1/2) pathways [5]. Activation of the TGF-β1 signaling pathway induces fibroblast proliferation, migration, and fibroblast-to-myofibroblast transition (FMT). TGF-β1 induces myofibroblasts to excessively secrete extracellular matrix (ECM) components, including collagen I and fibronectin, ultimately leading to collagen deposition [6,7]. It also upregulates matrix metalloproteinases (MMPs) that selectively degrade ECM in lung tissue, further exacerbating PF [8]. Thus, inhibiting myofibroblast activation by targeting TGF-β1 may be an effective strategy for preserving normal lung structure in the context of PF.

Traditional Chinese medicine has always been a valuable source of drugs, whether for the discovery of leading compounds or direct use as therapeutic agents. *Artemisia rupestris* L. is widely distributed in Xinjiang, China. As part of traditional Chinese medicine (TCM), *Artemisia rupestris* L. contains multiple chemical components, including flavonoids, organic acids, and sesquiterpenes [9,10]. These active constituents have demonstrated significant anti-inflammatory, antioxidant, and immunomodulatory activities [11,12]. Studies have shown that flavonoid compounds can treat PF [13], and suppressing TGF-β1 induces myofibroblast activation and reduces the production of ECM, thereby alleviating PF [14,15]. Therefore, investigating whether *Artemisia rupestris* L. has antifibrotic properties and identifying its potential treatment components has important significance for the discovery of PF treatments.

In the present research, we investigated the therapeutic effects and mechanisms of action of *Artemisia rupestris* L. ethanol extract (ER) against cigarette smoke (CS)-induced PF and isolated the active flavonoid compounds that treat PF by targeting TGF-β1. Using in vitro and in vivo experiments, we confirmed that the ER suppressed fibroblast activation and FMT via the TGF-β1 signaling pathway and decreased collagen production, thus treating PF. We used network pharmacology and molecular docking to screen potential active components and, combined with LC–MS and pull-down assays, we identified the active ingredient in ER that binds to TGF-β1 as Luteolin 7-glucuronide (LG). LG inhibited fibroblast activation and FMT, while increasing E-cadherin expression to enhance fibroblast adhesion via the TGF-β1 signaling pathway. Furthermore, LG alleviated PF by decreasing collagen I production, thus inhibiting collagen deposition.

## 2. Materials and Methods

### 2.1. Reagents

Luteolin 7-glucuronide (LG, C_21_H_18_O_12_, MW: 462.36, >99.8% purity) was purchased from Derick Biotechnology company (Chengdu, China). Dexamethasone (DEX, C_22_H_29_FO_5_, MW: 392.46, >97% purity) was purchased from Sigma-Aldrich (Cat# HY-14648; Saint Louis, MO, USA). Mouse TNF-α ELISA Kit (Cat#E-EL-M0044, Elabscience, Wuhan, China), Mouse IL-6 ELISA Kit (Cat#E-EL-M3063, Elabscience, China), and Mouse IL-10 ELISA Kit (Cat#E-EL-M0046, Elabscience, China) were purchased from Elabscience; TGF-β1 proteins were purchased from SinoBiological (Cat#10804-HNAC; Beijing, China); superoxide dismutase (SOD), catalase (CAT), and glutathione (GSH) kits were purchased from Solarbio (Cat#BC5165, Cat#BC0205, Cat#BC1175; Beijing, China); and rProtein A/G kit was purchased from Yeasen (Cat#36421ES40; Shanghai, China).

### 2.2. Drug Preparation and Extraction

*Artemisia rupestris* L. was collected at Balikun prairie in Xinjiang province, China, and identified by Prof. Li Baoli. A voucher specimen (No. 2018-17) was deposited in the Institute of Medicinal Plant Development, Beijing, China. A 100 g mass of *Artemisia rupestris* L., except roots, was extracted using 1 L of hot 80% ethanol under reflux for 2 h at 80 °C. This was repeated three times. The 3 L of extract was concentrated to 100 mL (1 g/mL). Subsequently, freeze-drying was performed using a lyophilizer, and the ER was stored at −80 °C.

### 2.3. Animals

Animal experiments were performed according to ethical policies and procedures approved by the Institutional Animal Care and Use Committee of China Agriculture University, Beijing, China (Approval number NO.AW72403202-2-1). Male C57BL/6 mice (weight: 20–22 g; age: 6–8 weeks) were purchased from SPF biotechnology company (Beijing, China) and raised under standard specific pathogen-free conditions of 23 ± 2 °C and 12/12 h light/dark cycles, and given standard animal chow and water. The mice were randomly assigned into seven groups (*n* = 7 per group): (a) control group, (b) CS group, (c) CS + ER-L (125 mg/kg) group, (d) CS + ER-H (500 mg/kg) group, and (e) CS + DEX (2.5 mg/kg) group. Mice in the CS and CS + ER groups were placed in a homemade smoke box (40 × 30 × 25 cm) with a three-way valve to draw the smoke and guide it into the box. Commercial cigarette-removed filters, comprising 10 mg of tar, 11 mg of carbon monoxide, and 1.0 mg of nicotine per cigarette, were used in the study. The animals stimulated by CS were placed in one box, received CS from 12 cigarettes per day, 6 days/week, for a total of 10 weeks. The methods were adapted from a previous study [16]. ER was dissolved in normal saline and administered to the mice 1 h before CS exposure.

### 2.4. Histopathological Observation

Lung tissues from identical regions in the mice were fixed in 4% formaldehyde, paraffin-embedded, and sectioned into 4 μm slices. To evaluate pulmonary histoarchitecture, tissue sections were stained with hematoxylin and eosin (H&E). Collagen and elastic fibers were visualized using Masson’s trichrome (Masson) and Elastica van Gieson (EVG) staining, respectively. The blue-stained regions surrounding the airway were designated as positive areas and subsequently outlined for further analysis. The proportion of the positive area within the bronchial wall was calculated to quantify the extent of subepithelial fibrosis using the Image J software 1.54p.

### 2.5. Preparation of Cigarette Smoke Extract

Cigarette smoke extract (CSE) solution was prepared as previously described [17], but with some modifications. Briefly, CSE was prepared by burning commercial cigarettes without filters. The smoke was bubbled into 10 mL of PBS for 5 min, and CSE was standardized by measuring the absorbance (OD = 0.9–1.0) at a wavelength of 320 nm. The pH was adjusted to 7.0–7.5 and the extract filtered using a 0.22 μm syringe filter to remove bacteria and large particles. The concentration of CSE in the PBS was defined as 100%, and the prepared CSE was used within 30 min of collection.

### 2.6. Cell Culture and Treatment

L929 cells were obtained from Pricella Biotechnology (Cat#CL-0137; Wuhan, China). and cultured in Dulbecco’s modified Eagle medium (DMEM, Cat#11965092; Thermo Fisher, Waltham, MA, USA) containing 10% fetal bovine serum (FBS, Cat#SE100-B; WISENT, Nanjing, China) and 1% penicillin–streptomycin. All cells were free from mycoplasma contamination and were grown at 37 °C in an atmosphere with 5% CO_2_. The freeze-dried ER powder was dissolved in DMEM to prepare a 40 mg/mL drug solution. This solution was filtered through a 0.22 μm filter (Cat#BS-PES-22; Biosharp, Hefei, China) to obtain the stock solution, which was diluted with DMEM to obtain a working solution. The drug treatment groups were pre-incubated with ER or LG for 1 h, and then co-incubated with 5% CSE or TGF-β1 (10 ng/mL) for 24 h. The experimental groups were grouped as follows: control group (0 mg/mL ER and 0% CSE), ER-H group (5 mg/mL ER and 0% CSE), CSE group (0 mg/mL ER and 5% CSE), CSEER-L (1.25 mg/mL ER and 5% CSE), CSEER-M (2.5 mg/mL and 5% CSE), CSEER-H (5 mg/mL and 5% CSE), LG-H (50 ug/mL LG and 0 ng/mL TGF-β1), TGF (0 ug/mL LG and 10 ng/mL TGF-β1), TGFLG-L (12.5 ug/mL LG and 10 ng/mL TGF-β1), TGFLG-M (25 ug/mL LG and 10 ng/mL TGF-β1), and TGFLG-H (50 ug/mL LG and 10 ng/mL TGF-β1).

### 2.7. Total RNA Extraction and Real-Time Quantitative PCR

Total RNA was isolated using TRIzol (Cat#R1100; Solarbio, Beijing, China), and the RNA concentrations were measured using a Nanodrop spectrophotometer (Thermo Fisher, Waltham, MA, USA). cDNA synthesis was performed using RevertAid First Strand cDNA Synthesis Kit (Cat#K1622; Thermo Fisher, Waltham, MA, USA). RT-qPCR was carried out on the CFX96 Real-Time PCR Detection System (Bio-Rad, Hercules, CA, USA) using SYBR Green Master Mix (Cat#Q712-02; Vazyme Biotech, Nanjing, China). The primers used are presented in Table S1.

### 2.8. Biochemical Index

Lung tissue samples were analyzed to measure TNF-a, IL-6, IL-10, superoxide dismutase (SOD), catalase (CAT), and glutathione (GSH). TNF-α, IL-6, and IL-10 detection was performed using enzyme-linked immunosorbent assay (ELISA). All biochemical assays were performed according to the kit instructions provided by the supplier.

### 2.9. Immunofluorescence (IF) Staining

The L929 cells were seeded in a 24-well plate as previously described [18]. At the end of the treatment, cells were fixed with paraformaldehyde (Cat#P1110; Solarbio, Beijing, China) for 15 min at room temperature and washed three times with PBS. Antigen retrieval from the lung sections was conducted using 5% bovine serum albumin (BSA) blocking. The cells and sections were cultured overnight with the primary antibody. Primary antibodies against α-SMA (1:100; Cat#67735-1-Ig; Proteintech, Wuhan, China), collagen I (1:200; Cat#bs-0578R; Bioss, Beijing, China), and MMP-2 (1:100; Cat#ab92536; Abcam, Cambridge, MA, USA) were prepared in advance. Finally, observations were made under a fluorescence microscope for image acquisition. The mean fluorescence intensity (MFI) of the images was determined using Image J 1.54p.

### 2.10. Cell Viability Assay

An MTT assay was used to evaluate drug cytotoxicity. Briefly, L929 cells were added to 96-well plates at a concentration of 5 × 10^3^ cells per well and maintained under culture conditions at 37 °C overnight. After treatment with drugs for 24 h, the cells were exposed to MTT solution for an extra 4 h at 37 °C. Afterward, the supernatant was discarded, and 150 μL of DMSO was dispensed into each well. A microplate reader (Tecan, Mannedorf, Switzerland) was used to measure absorbance at 490 nm. The average absorbance of three independent experiments, each consisting of three replicates, was calculated.

### 2.11. Wound Healing Assay

L929 cells were seeded in a 6-well plate and cultured for 24 h. A scratch wound assay was performed using a 200 μL pipette tip. The scratches were observed under a microscope (Leica, Solms, Germany) after the cells were treated with drugs. Image J software was used to determine the widths of the scratches.

### 2.12. Western Blot

Protein lysis buffer containing inhibitors was added to the sample in each group, and then the sample was incubated for 10 min at 4 °C. The supernatant was collected after centrifugation (10 min, 12,000 rpm, 4 °C). BCA kits (Cat#CW0014S; Cowin Biotech, TaiZhou, China) were used to determine protein concentration. Protein samples were separated using SDS-PAGE and transferred onto a PVDF membrane. The membrane was incubated with the homologous antibodies and tested using enhanced ECL reagents (Cat#PK10003; Proteintech, Wuhan, China). The corresponding primary antibodies were diluted with the blocking solution as follows: GAPDH (Cat#60004-1-Ig; Proteintech, Wuhan, China; 1:5000), Collagen I (Cat#66761-1-Ig; Proteintech, Wuhan, China; 1:1000), α-SMA (Cat#67735-1-Ig; Proteintech, Wuhan, China; 1:2000), MMP-2 (Cat#66366-1-Ig; Proteintech, Wuhan, China; 1:1000), p-Smad2/3 (Cat#8828; Cell Signaling Technology, Danvers, MA, USA; 1:1000), Smad2/3 (Cat#ab202445; Abcam, Cambridge, MA, USA; 1:2000), MMP-9 (Cat#ab76003; Abcam, Cambridge, MA, USA; 1:1000), TGF-β1 (Cat#ab315254; Abcam, Cambridge, MA, USA; 1:2000), ERK1/2 (Cat#11257-1-AP; Proteintech, Wuhan, China; 1:1000), and P38 (Cat#14064-1-AP; Proteintech, Wuhan, China; 1:1000). The proteins were then incubated with the corresponding HRP-labeled secondary antibody (Cat#A21020; Abbkine scientific, Wuhan, China; 1:5000). All antibodies were diluted with 5% BSA. Quantitative analysis of protein bands was conducted using Image J software.

### 2.13. Flow Cytometry

L929 cells were grown in 6-well plates for 24 h at a density of 10 × 10^4^ cells/well as previously described [19] to quantify DNA in nuclei stained with propidium iodide (PI). After drug treatment, a 0.25% trypsin–EDTA solution was used to trypsinize the cell pellets. Centrifugation was used to harvest the cell pellets, which were then washed with PBS solution. The cell pellets were incubated in ice-cold 70% ethanol at 4 °C for 24 h and then washed three times with PBS solution. Subsequently, the cells were incubated with RNase A and PI (Solarbio, Beijing, China) for thirty minutes. Cell cycle distribution analysis was carried out using a CytoFLEX flow cytometer (Beckman Coulter, Brea, CA, USA).

### 2.14. Detection of Cell Proliferation Using EdU

Cell proliferation was assessed using the BeyoClick EdU-488 kit (Cat#C0071S; Beyotime, Shanghai, China) according to the manufacturer’s protocol, as previously described [20]. In brief, the L929 cells were pre-treated for 1 h using medium containing various drug concentrations, followed by co-incubation with TGF-β1 or CSE for 24 h. EdU-488 was added to the medium, resulting in a final concentration of 10 mM. The cells were then incubated for 2 h. The cells were incubated with the click reaction solution in the dark at room temperature for 30 min, then washed three times with TBST. The cells were then incubated with Hoechst 33,342 (diluted 1:1000) in the dark at room temperature for 10 min. After washing, they were observed under a fluorescence microscope for image acquisition. The MFIs of the images were determined using Image J 1.54p.

### 2.15. LC–MS Analysis

One hour after administering ER to the mice, their urine was collected. The samples were redissolved in 1 mL of 80% methanol, and then sonicated and centrifuged at 14,000 g for 10 min. Chromatographic separation was performed using the Q Exactive Plus Orbitrap system (Thermo) with an Acquity UPLC HSS T3 column (2.1 × 100 mm, 1.8 μm). The elution process occurred at a flow rate of 0.3 mL/min using 0.1% formic acid in water and 0.1% formic acid in acetonitrile. Data analysis was performed using the Compound Discoverer v3.3 software, and compounds were identified using local and online mzCloud databases.

### 2.16. Network Pharmacology

Potential active ingredients in ER were identified based on LC–MS detection of natural components in urine. The Traditional Chinese Medicine Database and Analysis Platform (TCMSP) (https://old.tcmsp-e.com/tcmsp.php; accessed on 12 November 2024) was used to identify the targets of active compounds. PF targets were searched in Drugbank, GeneCards, and Online Mendelian Inheritance in Man (OMIM) databases. Finally, a network of drug genes was constructed to extract the IDs of the overlapping genes in the databases. The overlapping targets were input into the online Database for Annotation, Visualization, and Integrated Discovery (DAVID), and enriched candidate pathways were further analyzed to identify associated biological processes.

### 2.17. Molecular Docking

The 3D structures of proteins (MAPK14, PDB ID: 7BE5; EGFR, PDB ID: 1XKK; MAPK1, PDB ID: 1TVO; MAPK3, PDB ID: 2ZOQ; MMP-2, PDB ID: 7XGJ; MMP-9, PDB ID: 1GKC; TGF-β1, PDB ID: 1KLA) were obtained from the Protein Data Bank (https://www.rcsb.org/, accessed on 15 December 2024). The 3D structure of the small molecule was downloaded from the PubChem database (https://pubchem.ncbi.nlm.nih.gov/, accessed on 15 December 2024). Prior to docking, the receptor protein was pre-processed using the Molecular Operating Environment (MOE, Version 2022.02) software, and water molecules, salt ions, and small molecules were removed. Docking of chemical constituents with protein was conducted using the MOE software, and a docking score was calculated.

### 2.18. Pull-Down Assay

To perform the pull-down assay, L929 cells were pre-treated with 10 ng/mL of TGF-β1 for 24 h, and then collected and lysed using RIPA buffer containing protease and phosphatase inhibitors. The primary antibody targeting TGF-β1 (1:30; Cat#bsm-33287M; Bioss, Beijing, China) was reacted with protein A/G magnetic beads and incubated overnight at 4 °C. The antibody–bead complexes were then mixed with whole-cell lysates and incubated overnight at 4 °C. Subsequently, the protein mixture was separated using a magnetic rack, and the precipitates were washed three times with TBS solution. The precipitates were then incubated with 5 mg/mL of ER for 1 h to isolate target compounds. After incubation, the precipitates were washed three times with TBS solution. The active target components were eluted from Protein A/G magnetic beads using 0.1% (*v*/*v*) aqueous formic acid and then subjected to LC–MS analysis.

### 2.19. Surface Plasmon Resonance (SPR) Analysis

The binding affinity of LG to TGF-β1 was measured using Biacore T200 (Cytiva, Marlborough, MA, USA) with a CM5 sensor chip (GE Healthcare, Marlborough, MA, USA). Solutions of compounds were prepared in a running buffer by serial dilution of stock solutions. Subsequently, different concentrations of LG (3.125–200 μg/mL) were diluted in running buffer and injected into the system as the analyte. The parameters for SPR were set as follows: flow rate, 30 μL/min; association time, 90 s; dissociation time, 90 s; temperature, 25 °C. The interaction parameters were obtained using the Biacore evaluation software (Version 1.0).

### 2.20. CETSA Assay

L929 cells were cultured overnight in 100 mm dishes. RIPA buffer (Cat#R0010; Solarbio, Beijing, China) was used to obtain cellular proteins, and BCA kits were used to quantify protein content. The proteins were diluted to 5 μg/μL before drug treatment. The proteins were mixed with LG (50 μg/mL) and rotated for 1 h at room temperature. The proteins were incubated at specific temperatures (51 °C, 54 °C, 57 °C, 60 °C, 63 °C, 66 °C, 69 °C, 72 °C) for 3 min. Western blot analysis was performed on each sample.

## 3. Results

### 3.1. ER Attenuates CS-Induced Lung Inflammation, Oxidative Stress, and PF

**Figure 1:**
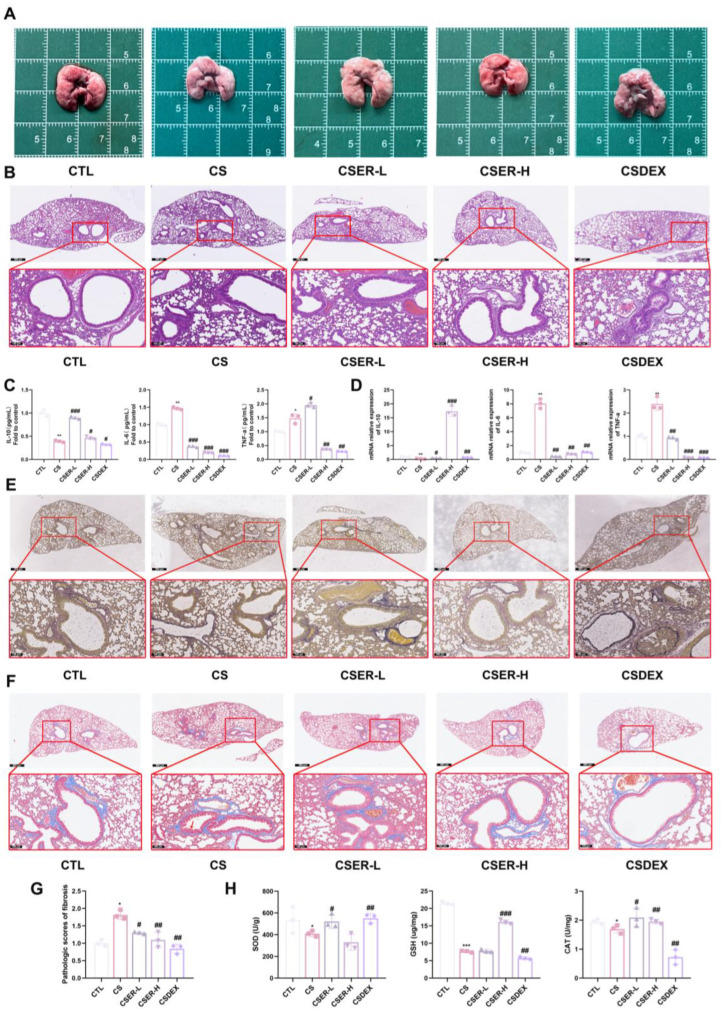
ER improved the lung structure of mice with CS-induced lung inflammation, oxidative stress, and fiber imbalance. The drugs were administered to the mice orally 1 h before CS exposure, and the same procedures were followed for the experiments. Representative images of lung tissue from mice in each group (**A**). Representative images (scale bars = 500 μm and 100 μm) of hematoxylin and eosin (H&E)-stained lung tissue slides (**B**). ELISA for IL-10, IL-6, and TNF-α in lung tissue (**C**). qRT-PCR assay for mRNA expression of *IL-10*, *IL-6*, and *TNF-α* in lung tissue (**D**). Representative images (scale bars = 100 μm and 500 μm) of EVG-stained lung tissue slides (**E**). Representative images (scale bars = 100 μm and 500 μm) of Masson’s trichrome-stained lung tissue slides (**F**). Quantitative analysis of blue-stained collagen expression in lung tissue (**G**). Assays for SOD, GSH, and CAT levels in lung tissue (**H**). The results shown are mean ± SD (*n* = 3). Student’s *t*-test was used for two-group comparisons. * *p* < 0.05, ** *p* < 0.01, *** *p* < 0.001 compared with CTL. # *p* < 0.05, ## *p* < 0.01, ### *p* < 0.001 compared with CS.

The analysis of the mouse lung tissue showed a rougher surface and relatively stiffer texture in the CS-induced lung tissue. ER demonstrated therapeutic efficacy against the observed pathology; however, DEX treatment caused significant pulmonary atrophy, indicative of adverse effects (Figure 1A). The H&E staining results showed that ER improved CS-induced inflammatory cell infiltration, alveolar wall thickening, and airway remodeling (Figure 1B). To characterize the protective effect of ER against lung inflammation in CS-induced mice, the expression of the inflammatory and anti-inflammatory markers IL-10, IL-6, and TNF-α was evaluated using qRT-PCR and ELISA. The results showed that ER inhibited the production of the inflammation factors IL-6 and TNF-α in the lungs, and increased the production of the anti-inflammation factor IL-10 (Figure 1C,D). The EVG staining indicated that ER reduced CS-induced collagen deposition and alleviated abnormal elastic fiber (shortening and thickening) (Figure 1E). Furthermore, Masson’s trichrome staining demonstrated that ER effectively inhibited CS-induced collagen deposition in and around the airway (Figure 1F,G). At the same time, ER improved CS-induced SOD, GSH, and CAT imbalance in lung tissue (Figure 1H). In conclusion, ER can inhibit CS-induced pulmonary inflammation and oxidative stress, improving the imbalance in fibrous distribution that results in pulmonary fibrosis, thereby attenuating CS-induced lung damage.

### 3.2. ER Inhibits CS-Induced Collagen Deposition and MMP Secretion to Prevent PF

**Figure 2:**
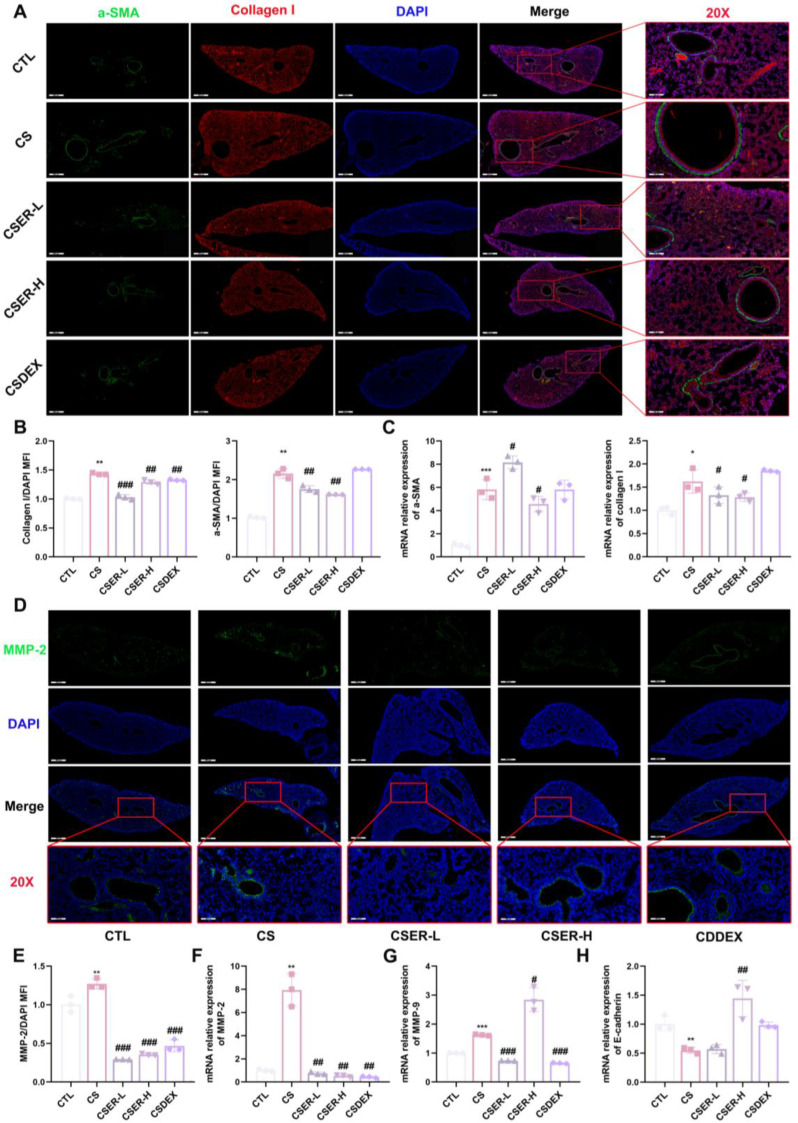
ER inhibits CS-induced collagen deposition and MMP secretion. Representative images (scale bars = 100 μm and 400 μm) of collagen I (red), α-SMA (green), and DAPI (blue) IF staining (**A**). Quantification of collagen I and α-SMA expression in lung tissues (**B**). qRT-PCR assay for mRNA expression of *collagen I* and *α-SMA* in lung tissue (**C**). Representative images (scale bars = 100 μm and 400 μm) of MMP-2 (green) and DAPI (blue) IF staining (**D**). Quantification of MMP-2 expression in lung tissues (**E**). qRT-PCR assay for mRNA expression of *MMP-2* (**F**), *MMP-9* (**G**), and *E-cadherin* (**H**) in lung tissue. The results shown are mean ± SD (*n* = 3). Student’s *t*-test was used for two-group comparisons. * *p* < 0.05, ** *p* < 0.01, *** *p* < 0.001 compared with CTL. # *p* < 0.05, ## *p* < 0.01, ### *p* < 0.001 compared with CS.

PF is the most significant cause of lung structure injury [21]. The specific markers of α-SMA and collagen I proteins were labeled using IF and then used to evaluate the anti-PF effect of ER. α-SMA and collagen I proteins were marked with green and red fluorescence, respectively (Figure 2A). As shown in Figure 2B, ER alleviated the pathological changes in α-SMA and collagen I in the pulmonary airway and tissue, respectively. Furthermore, the levels of *α-SMA* and *collagen I* mRNA in the lung tissue were inhibited by ER treatment (Figure 2C). Additionally, CS upregulated MMP-2 expression, which selectively degraded elastic fibers, leading to collagen deposition and uneven distribution of elastin fibers in the lungs [22]. However, ER inhibited the increased CS-induced MMP-2 protein secretion in the airway and lung tissue (Figure 2D,E) and significantly inhibited the up-regulation of *MMP-2* and *MMP-9* mRNA expression (Figure 2F,G). Moreover, ER inhibited the increase in *E-cadherin* mRNA expression levels associated with CS-induced PF (Figure 2H). Thus, ER inhibits CS-induced secretion of collagen I protein and MMPs, alleviating PF.

### 3.3. ER Inhibited CSE-Induced Fibroblast Activation

**Figure 3:**
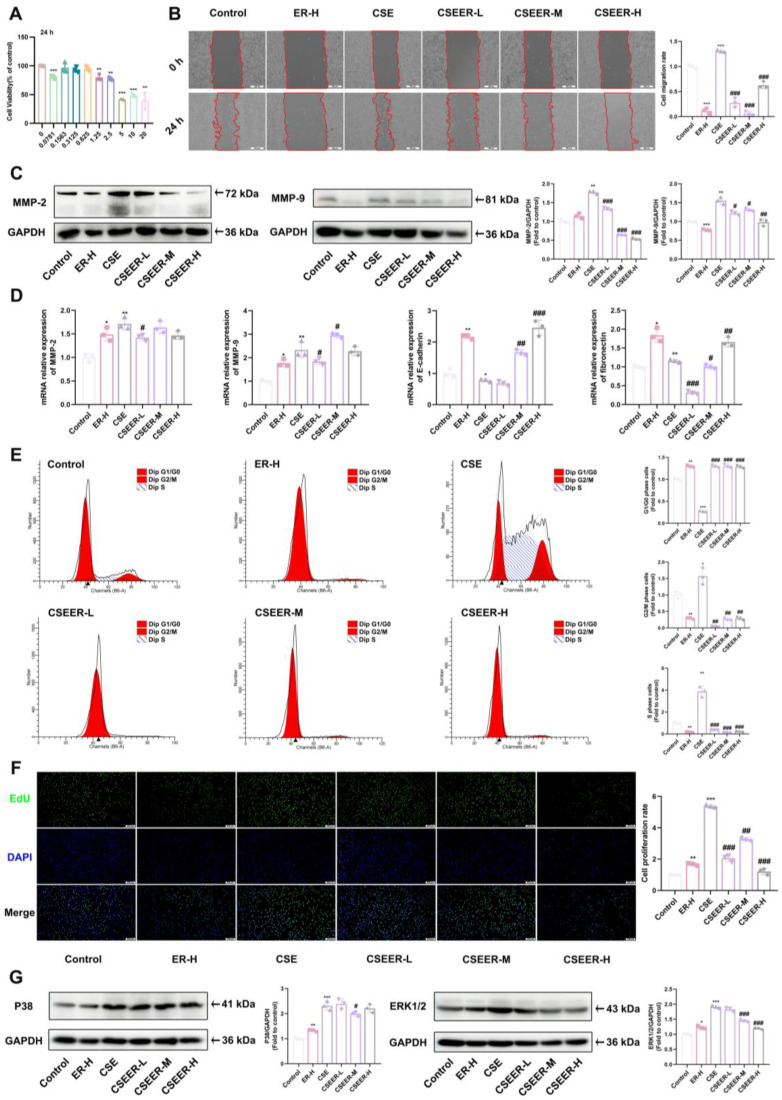
ER inhibited CSE-induced fibroblast activation. L929 cells were pre-incubated with ER for 1 h, and then co-incubated with 5% CSE for 24 h. (**A**) The effect of ER on L929 cell viability was assayed using MTT. Representative images (scale bar = 500 μm) of wound healing in L929 cells and the cell migration rate (**B**). Expression of MMP-2 and MMP-9 was determined using Western blot analysis (**C**). qRT-PCR assay for *MMP-2*, *MMP-9*, *E-cadherin*, and *fibronectin* mRNA expression in L929 cells (**D**). Representative images and statistics of the cell cycle distribution of L929 cells (**E**). The proliferation of L929 cells was determined using the EdU staining kit (scale bar: 200 μm) (**F**). Expression of P38 and ERK1/2 proteins was determined using Western blot analysis (**G**). The results shown are mean ± SD (*n* = 3). Student’s *t*-test was used for two-group comparisons. * *p* < 0.05, ** *p* < 0.01, *** *p* < 0.001 compared with control. # *p* < 0.05, ## *p* < 0.01, ### *p* < 0.001 compared with CSE.

In order to investigate the effect of ER on PF, we established an in vitro model using CSE-treated L929 cells. The L929 cells were treated with a series of ER concentrations ranging from 0 to 20 mg/mL. ER at concentrations of 1.25, 2.5, and 5 mg/mL inhibited the viability of the L929 cells (Figure 3A). In the wound healing experiments, ER significantly inhibited CSE-induced L929 cell migration (Figure 3B). MMP-9 and MMP-2 proteins, two types of MMPs, were degraded in the ECM, promoting L929 cell migration [23]. However, ER inhibited the CSE-induced increase in MMP-9 and MMP-2 (Figure 3C) and the increase in the mRNA expression of migration-related *MMP-2*, *MMP-9*, and *fibronectin* while increasing the mRNA level of *E-cadherin*, thereby enhancing the adhesion of CSE-induced L929 cells. Meanwhile, CSE significantly promoted cell cycle progression in the L929 cells from the G1/G0 phase to the S and G2/M phases, thereby accelerating proliferation. In contrast, ER arrested the cell cycle of the L929 cells at the G1/G0 phase, consequently inhibiting proliferation (Figure 3E). Using EdU assays, we observed that ER inhibited the CSE-induced proliferation of the L929 cells (Figure 3F). P38 and ERK1/2 proteins are key targets in the regulation of the cell cycle [24]. The Western blot analysis showed that the expression of P38 and ERK1/2 proteins was significantly decreased in the ER-treated L929 cells (Figure 3G). Therefore, ER inhibits CSE-induced cell proliferation and migration, regulates cell cycle progression, and suppresses L929 cell activation.

### 3.4. ER Inhibits CSE-Induced FMT and Collagen Deposition via the TGF-β1 Signaling Pathway

**Figure 4:**
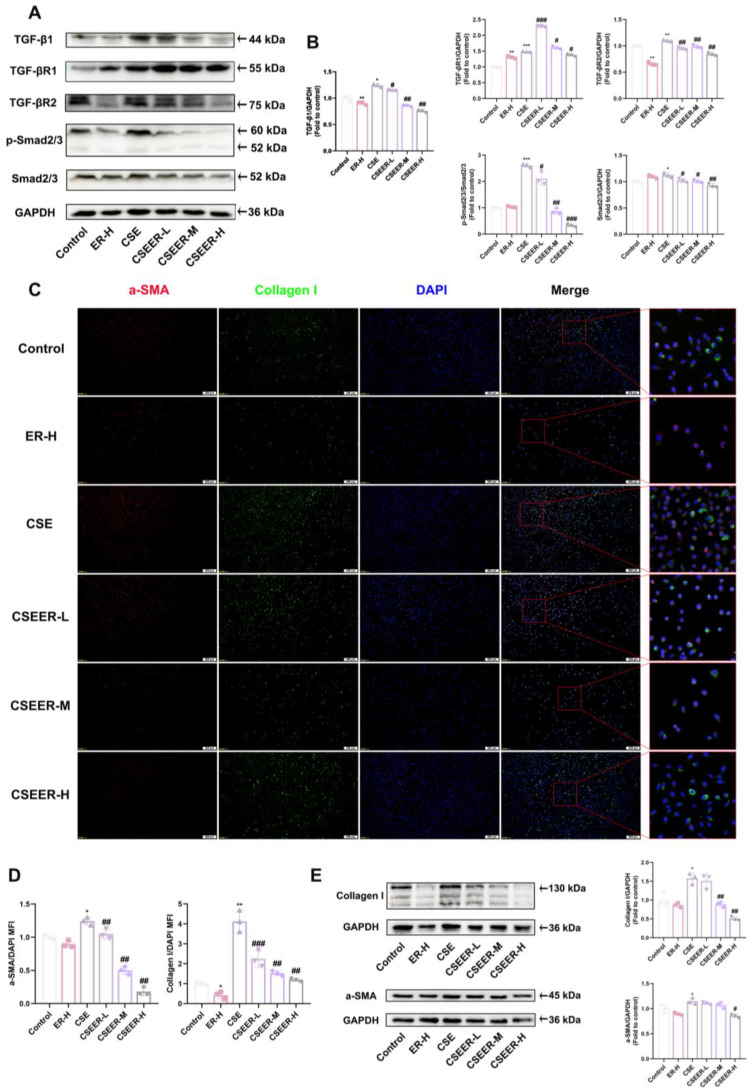
ER inhibits CSE-induced FMT and collagen deposition via the TGF-β1 signaling pathway. TGF-β1, TGF-βR1, TGF-βR2, p-Smad2/3, and Smad2/3 protein levels in L929 cells were determined using immunoblotting analysis (**A**). Quantification of TGF-β1, TGF-βR1, TGF-βR2, p-Smad2/3, and Smad2/3 proteins using Western blots (**B**). Representative IF images (scale bar = 200 μm) of collagen I (green), α-SMA (red), and DAPI (blue) expression in L929 cells (**C**). Quantification of collagen I and α-SMA expression in L929 cells (**D**). Collagen I and α-SMA protein levels in L929 cells were assessed using Western blotting (**E**). The results shown are mean ± SD (*n* = 3). Student’s *t*-test was used for two-group comparisons. * *p* < 0.05, ** *p* < 0.01, *** *p* < 0.001 compared with control. # *p* < 0.05, ## *p* < 0.01, ### *p* < 0.001 compared with CSE.

TGF-β1 was identified as a key protein inducing FMT [25]. In the Western blot assays, CSE upregulated the expression of TGF-β1 and its downstream signaling pathway proteins, including TGF-βR1, TGF-βR2, p-Smad2/3, and Smad2/3. However, ER reversed this effect by inhibiting the increase in TGF-β1 protein (Figure 4A,B). As expected, the fluorescence intensities of collagen I and α-SMA in the CSE-induced L929 cells showed a negative correlation with the ER dose (Figure 4C,D). In the Western blot assay, the α-SMA and collagen I levels significantly increased after stimulation with CSE, but the protein levels decreased in the group treated with ER (Figure 4E). Therefore, ER inhibits CSE-induced upregulation of TGF-β1 expression, suppresses the TGF-β1 signaling pathway activation that promotes α-SMA and collagen I protein expression, and consequently prevents FMT and collagen deposition.

### 3.5. Screening for Active Components in ER Using Network Pharmacology and Molecular Docking

**Figure 5:**
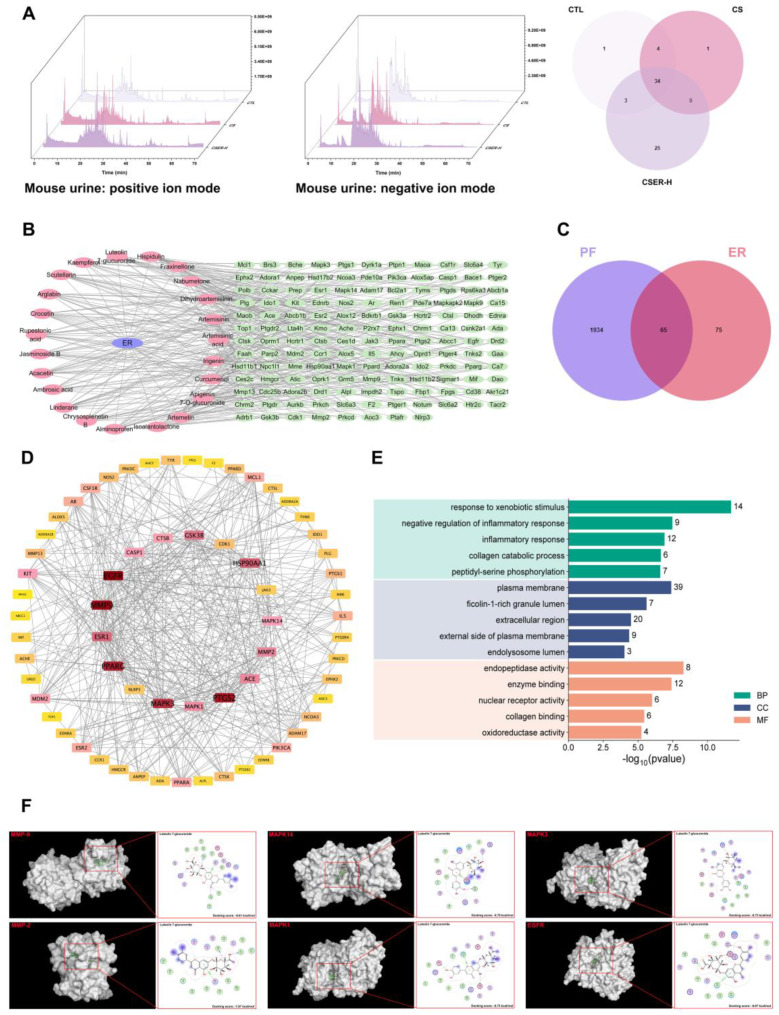
Screening for active components using network pharmacology and molecular docking. The TICs of the mice’s urine samples in positive and negative ion modes, along with the number of natural chemical constituents (**A**). The component–target network (**B**). Venn diagram of the active components and PF targets (**C**). PPI network of the 65 potential therapeutic targets (**D**). Results of GO term enrichment analysis: BP, biological process; MF, molecular function; CC, cellular component (**E**). Molecular docking of LG with MMP-9 (1GKC), MMP-2 (7XGJ), MAPK1 (1TVO), MAPK3 (2ZOQ), MAPK14 (7BE5), and EGFR (1XKK) using the MOE software (**F**).

To identify the active components in ER, the natural components in the urine of mice in the CTL, CS, and CSER-H groups were screened using LC–MS. These components were detected in both the positive and negative ion modes in the total ion current (TIC) plots (Figure 5A). Comparing the CSER-H group with the CTL and CS groups identified 25 active components (Table S2). Using Swiss Target Prediction, the targets of 23 active components were analyzed, and 140 potential targets were identified. In addition, 1999 targets associated with PF were identified in the Genetic Association Database. From these two lists, 65 of the therapeutic targets were identified by the intersection of potential therapeutic targets and PF targets (Figure 5C). The central protein–protein interaction (PPI) network was constructed using data from the STRING database, and the resulting network was imported into Cytoscape version 3.8.0 for further analysis. Potential therapeutic targets were imported into the STRING database to obtain PPI information and identify candidate targets within the PPI (Figure 5D). These results confirmed that MMP-9, MMP-2, MAPK14, MAPK1, and MAPK3 are key targets in ER treatment of PF. A GO-based bioinformatics analysis was performed to evaluate the network in terms of biological process (BP), cellular component (CC), and molecular function (MF) (Figure 5E). Additionally, the docking results revealed that Luteolin 7-glucuronide (LG) exhibited the lowest binding energy with potential targets, indicating its strongest binding affinity (Figure 5F). Thus, LG is a potential active compound in ER that can be used to treat PF.

### 3.6. LG Inhibits the TGF-β1 Signaling Pathway via Targeted Binding to TGF-β1

**Figure 6:**
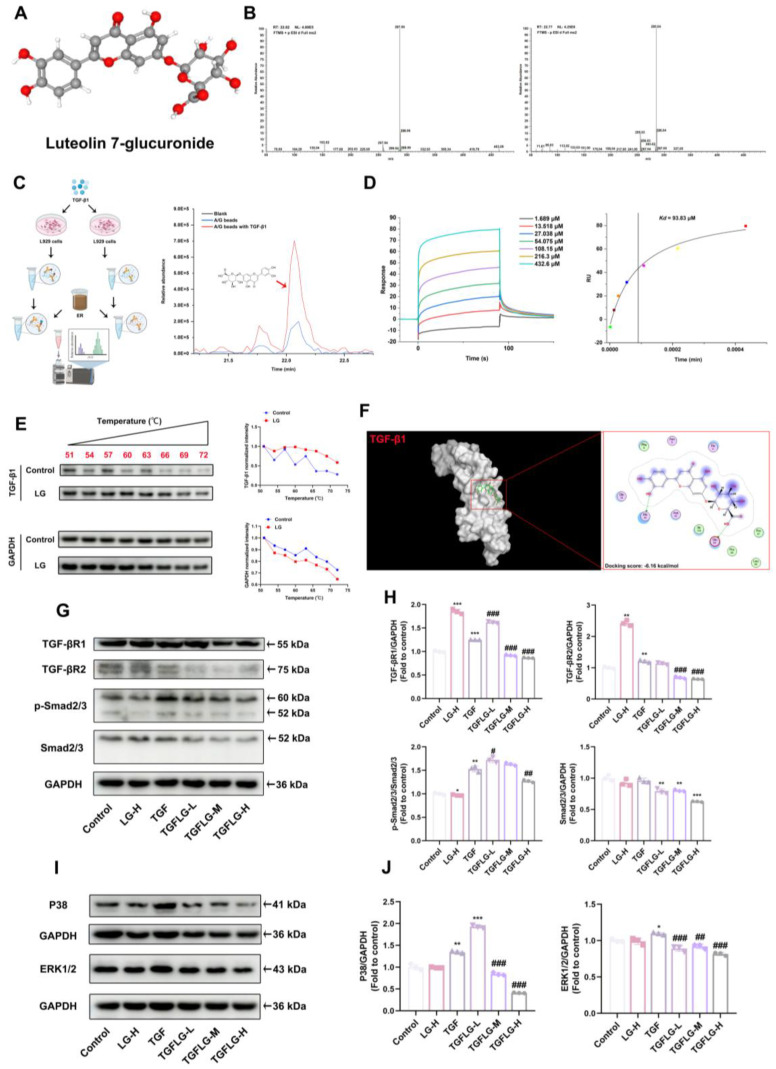
LG inhibits the TGF-β1 signaling pathway by targeted binding to TGF-β1. The chemical structure of LG (**A**). The MS2 fragments of the LG standard in positive ion mode and negative ion mode (**B**). Screening of TGF-β1-binding active ingredients in ER (**C**). SPR titration curve and dose–response plot of LG–TGF-β1 binding (**D**). Analysis of the binding affinity of LG for TGF-β1 was determined using the CETSA assay (**E**). Molecular docking of LG to TGF-β1 (1KLA) using MOE software (**F**). Representative Western blots for TGF-βR1, TGF-βR2, p-Smad2/3, and Smad2/3 protein expression and quantification of TGF-βR1, TGF-βR2, p-Smad2/3, and Smad2/3 Western blots (**G**,**H**). Representative Western blots for p38 and ERK1/2 protein expression and quantification of p38 and ERK1/2 Western blots (**I**,**J**). The results shown are mean ± SD (*n* = 3). Student’s *t*-test was used for two-group comparisons. * *p* < 0.05, ** *p* < 0.01, *** *p* < 0.001 compared with control. # *p* < 0.05, ## *p* < 0.01, ### *p* < 0.001 compared with TGF.

LG was identified using LC–MS, with MS2 fragmentation producing major fragments at 287.05 ([M + H], *m*/*z*) and 285.04 ([M − H], *m*/*z*). MS2 was subsequently used for its identification (Figure 6A,B). Pull-down and LC–MS assays were used to screen and identify compounds that bind to TGF-β1 in ER. The results showed that the active ingredient in ER that bound to TGF-β1 was LG (Figure 6C). The SPR analysis showed that LG (1.689–432.6 μM) directly interacted with TGF-β1 in a concentration-dependent manner, with an equilibrium dissociation constant (*Kd*) of 93.83 μM (Figure 6D). The CETSA analysis demonstrated that LG significantly protected the TGF-β1 protein from temperature-dependent denaturation, confirming that LG directly binds to the TGF-β1 protein (Figure 6E). In addition, the molecular docking predictions showed that LG bound to the His40 and Glu84 residues of the TGF-β1 protein (PDB: 1KLA), with a binding energy score of -6.16 kcal/mol (Figure 6F). To investigate the effects of LG on the TGF-β1 downstream signaling pathways, including both non-Smad and Smad pathways, the L929 cells were treated with 12.5, 25, and 50 μg/mL of LG and 10 ng/mL of TGF-β1 based on the SPR results. LG significantly inhibited TGF-β1-induced upregulation of TGF-βR1 and TGF-βR2 protein expression in the L929 cells, thereby suppressing the conversion of Smad2/3 to p-Smad2/3 (Figure 6G,H). Additionally, LG attenuated the TGF-β1-induced upregulation of P38 and ERK1/2 protein expression (Figure 6I,J). Therefore, LG inhibits both the Smad and non-Smad signaling pathways through targeted binding to TGF-β1.

### 3.7. LG Inhibits TGF-β1-Induced Fibroblast Activation and FMT

**Figure 7:**
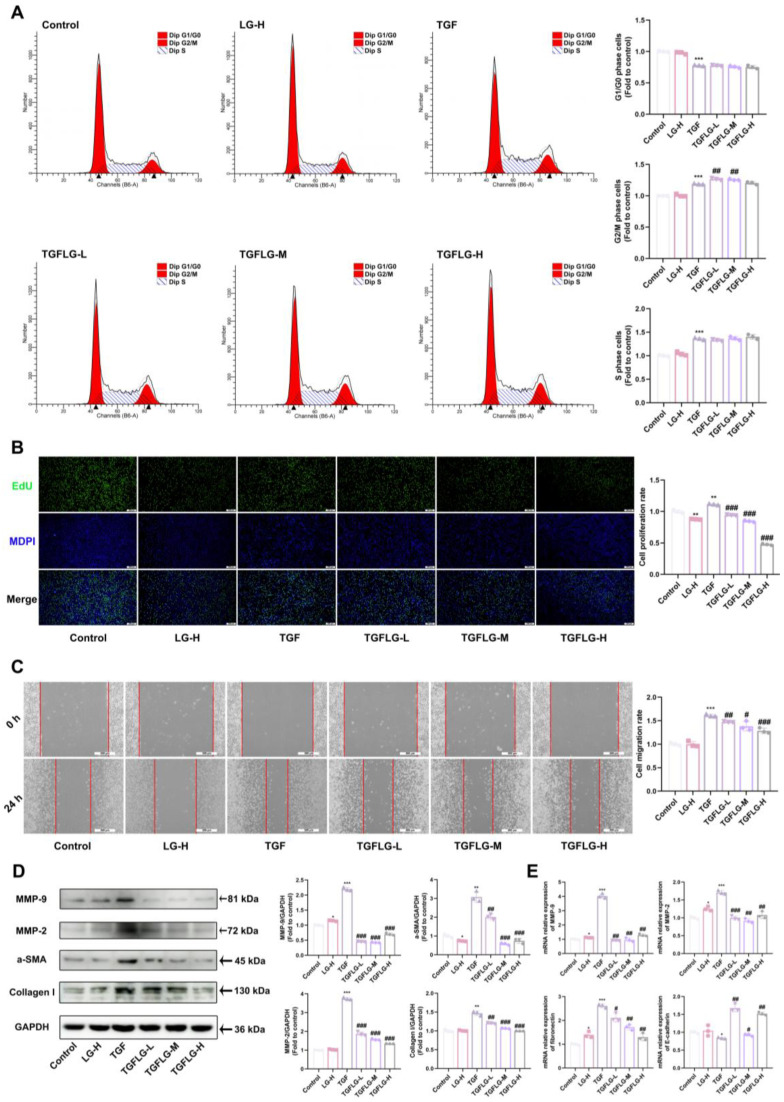
LG inhibits TGF-β1-induced fibroblast activation and FMT. Representative images and statistics of the cell cycle distribution of L929 cells (**A**). Proliferation of L929 cells was determined using the EdU staining kit (scale bar: 200 μm) (**B**). Representative images (scale bar = 500 μm) of wound healing in L929 cells and the cell migration rate (**C**). MMP-9, MMP-2, α-SMA, and collagen I protein levels in L929 cells were determined using immunoblotting analysis (**D**). qRT-PCR assay for *MMP-2*, *MMP-9*, *E-cadherin*, and *fibronectin* mRNA expression in L929 cells (**E**). The results shown are mean ± SD (*n* = 3). Student’s *t*-test was used for two-group comparisons. * *p* < 0.05, ** *p* < 0.01, *** *p* < 0.001 compared with control. # *p* < 0.05, ## *p* < 0.01, ### *p* < 0.001 compared with TGF.

The flow cytometry analysis revealed that TGF-β1 promotes cell cycle progression in L929 cells from the G1/G0 phase to the S and G2/M phases, while LG arrested L929 cells at the G2/M phase (Figure 7A). Using EdU assays, we showed that LG inhibited TGF-β1-induced proliferation of the L929 cells in a dose-dependent manner (Figure 7B). Meanwhile, LG inhibited TGF-β1-induced migration of the L929 cells (Figure 7C) and reduced the protein and mRNA expression levels of *MMP-9* and *MMP-2*, which promote cell migration (Figure 7D,E). LG inhibited TGF-β1-induced increases in the α-SMA and collagen I protein levels, suppressed *fibronectin* mRNA expression, and promoted *E-cadherin* mRNA expression in the L929 cells (Figure 7D,E). LG attenuates PF by inhibiting TGF-β1-induced activation and FMT in L929 cells, enhancing cellular adhesion, and reducing collagen I production.

## 4. Discussion

PF causes irreversible lung damage and severely threatens health. Chronic exposure to cigarette smoke is a major contributing factor to the pathogenesis of PF. Cigarette smoke induces sustained inflammatory and oxidative stress responses in the lungs, serving as an initiating factor for fibrosis development. Currently approved PF therapies can only slow the progression of the disease, failing to halt its advancement. Although numerous small molecule agents are under development, their clinical translation faces a protracted timeline. *Artemisia rupestris* L., a traditional Chinese herb with lung-clearing, lung-moistening, and anti-inflammatory properties, has never been investigated for its antifibrotic potential. In this study, we investigated the therapeutic effects of ER against CS-induced pulmonary inflammation, oxidative stress, and fibrosis.

PF usually occurs as the final stage of lung function damage, such as chronic obstructive pulmonary disease (COPD), lung inflammation, and so on. Persistent lung injury due to oxidative stress and chronic inflammation is a central feature of lung fibrosis [26,27]. In this study, we established a CS-induced mouse model to investigate the antifibrotic activity of ER and its potential as a new therapeutic for PF. The results showed that mice models of PF induced by CS have characteristics similar to human PF, such as airway remodeling, lung inflammation, fiber imbalance, and oxidative stress. ER significantly prevented inflammation, airway remodeling, oxidative stress, and collagen deposition, improving lung structure. However, DEX—a common treatment for lung inflammation and PF—had no significant protective effect on airway remodeling in the CS-exposed mice and exacerbated CS-induced pulmonary emphysema in the mice. Oxidative stress and chronic inflammation persistently activate myofibroblasts, promoting the secretion of collagen. Inhibiting the proliferation and activation of myofibroblasts is an important factor in the treatment of PF. This leads to the reduced secretion of collagen (such as collagen I) and MMPs, thereby attenuating collagen deposition in lung tissue [28,29]. EMT and FMT are critical factors driving the increase in myofibroblasts. Preventing the reduction in E-cadherin expression in cells, suppressing the upregulation of MMPs, and alleviating the imbalance in the ECM composition serve as key strategies for inhibiting EMT and FMT [30,31]. The in vivo experimental results demonstrated that CS induced a significant decrease in *E-cadherin* mRNA expression, suggesting that EMT could also contribute to CS-induced PF. The α-SMA protein expression as a marker of myofibroblasts was examined using an IF assay [32]. The results showed that ER inhibited the CS-induced increase in α-SMA protein expression around the airway, suppressing myofibroblast accumulation. Concurrently, the secretion of collagen I and MMP-2 decreased, along with a decrease in fibronectin mRNA, which alleviated collagen deposition in lung tissue. To further validate these findings, an in vitro model of L929 cells was used to demonstrate that ER significantly inhibited the CSE-induced activation and proliferation of L929 cells via the TGF-β1 signaling pathway, suppressed FMT and collagen deposition, and increased E-cadherin expression to enhance adhesion, thus confirming its antifibrotic effects.

To date, only two therapeutic agents—pirfenidone (targeting inflammasome pathways) and nintedanib (a tyrosine kinase inhibitor)—have been approved by international clinical regulators for the treatment of PF; thus, identifying and screening TCM extracts for chemical constituents that can be used for the treatment of PF is essential for single-chemical drug development. *Artemisia rupestris* L. mainly contains flavonoids, organic acids, and sesquiterpenes [33]. We used LC–MS to identify 25 active natural compounds in mouse urine. The potential targets of these active compounds against PF were predicted using network pharmacology and molecular docking. Based on network pharmacology, MMP-9, MMP-2, MAPK14, MAPK1, MAPK3, and EGFR were identified as key targets of active ingredients in ER for treating PF. The molecular docking simulations of the binding of the 25 active compounds with these key targets revealed LG as the compound exhibiting favorable binding to all key targets, consistent with the targets identified in the in vitro ER experimental results. LG was screened as a potential active compound for treating PF.

TGF-β1 is one of the most researched profibrotic cytokines and plays a central regulatory role in the pathogenesis of PF [34]. It regulates the activation of Smad and non-Smad downstream signaling pathways, inducing FMT and the activation of fibroblasts [35]. Targeting and binding the TGF-β1 protein to affect its function is an effective method for preserving normal lung structure in PF. Therefore, we performed pull-down and LC–MS testing to screen and identify compounds that bind to TGF-β1 in ER. We identified the active ingredient in ER that binds to TGF-β1 as LG. SPR and CETSA analyses were performed to quantify the interaction between LG and TGF-β1. In addition, a molecular docking analysis was carried out to elucidate the binding mode of LG and TGF-β1. LG interacted with His40 and Glu84 in TGF-β1. Finally, LG inhibits the activation of both Smad and non-Smad downstream signaling pathways by binding to TGF-β1; reverses the upregulation of α-SMA, collagen I, MMP-2, and MMP-9 protein expression; suppresses *fibronectin* expression; and increases *E-cadherin* expression to enhance fibroblast adhesion. Thus, it can suppress fibroblast activation and FMT to inhibit collagen deposition, hence treating PF.

## 5. Conclusions

This study deepened our understanding of how *Artemisia rupestris* L. prevents CS-induced PF and lung structural injury. It demonstrated that ER alleviates CS-induced PF by inhibiting fibroblast activation and FMT, thereby reducing collagen deposition. Through network pharmacology and molecular docking analyses, compounds in ER with potential activity against PF were screened. We identified the active compound in ER that binds to TGF-β1 as LG. LG inhibits the TGF-β1 signaling pathway through targeted binding to TGF-β1; reverses the upregulation of α-SMA, collagen I, MMP-2, and MMP-9 protein expression; and suppresses *fibronectin* expression while enhancing *E-cadherin* expression to promote fibroblast adhesion. Thus, it treats PF by suppressing fibroblast activation and FMT, ultimately inhibiting collagen deposition. In conclusion, *Artemisia rupestris* L. and its active compound LG represent potential clinical agents for the treatment of PF. These findings provide a foundational reference for future therapeutic drug development.

## Data Availability

The datasets generated/analyzed during this study are available upon request from the corresponding author.

## Supplementary Materials

The following supporting information can be downloaded at https://www.mdpi.com/article/10.3390/antiox14050533/s1: Table S1. Primers used for qPCR assays; Table S2. Mass spectrometry information of active components in urine.

## Abbreviations

The following abbreviations are used in this manuscript.

PF	pulmonary fibrosis
COPD	chronic obstructive pulmonary disease
TGF-β1	transforming growth factor beta 1
LC-MS	chromatograph-Mass Spectrometer
FMT	fibroblast-to-myofibroblast transition
ECM	extracellular matrix
CS	cigarette smoke
TCM	traditional chinese medicine
ER	*Artemisia rupestris* L. ethanol extract
LG	luteolin 7-glucuronide
DEX	dexamethasone
CSE	cigarette smoke extract
L929	NCTC clone 929
TIC	total ion-current
TGF-β1	transforming growth factor-β1
TGF-βR1	transforming growth factor β receptor 1
TGF-βR2	transforming growth factor β receptor 2
SMA	α-smooth muscle actin
DMEM	dulbecco’s modified eagle medium
Elisa	enzyme-linked immunosorbent assay
Masson	masson’s trichrome
EVG	elastica van gieson
MMPs	matrix metalloproteinases
IF	immunofluorescence
SPR	surface plasmon resonance
