# Supplementary material for: Luteolin 7-Glucuronide in Artemisia rupestris L. Extract Attenuates Pulmonary Fibrosis by Inhibiting Fibroblast Activation and FMT via Targeting of TGF-β1
Source: Antioxidants (Basel). 2025 Apr 29;14(5):533. doi: 10.3390/antiox14050533 (PMC12108481; doi:10.3390/antiox14050533)
Supplement: Supplementary file 1 [file antioxidants-14-00533-s001.zip › antioxidants-3569877-supplementary.pdf]

Supplementary Data

Luteolin 7-Glucuronide in *Artemisia rupestris* L. Extract Attenuates Pulmonary Fibrosis by Inhibiting Fibroblast Activation and FMT via Targeting of TGF-β1

Table S1. Primers used for qPCR assays.

Name	Primer	Sequence	Size
a-SMA	Forward	GCTGTTGTAGGTGGTCTCAT	99bp
	Reverse	ACCATCGGCAATGAGCGTTT	
Collagen I	Forward	GAGAGCGAGGCCTTCCCGGA	132bp
	Reverse	GGGAGCCAGCGGGACCTTGT	
Fibronectin	Forward	AAGGCTGGATGATGGTGGACT	140bp
	Reverse	TCGGTTGTCCTTCTTGCTCC	
MMP-9	Forward	GTCCAGACCAAGGGTACAGC	107bp
	Reverse	ATACAGCGGGTACATGAGCG	
MMP-2	Forward	GCCCCGAGACCGCTATGTCCACT	170bp
	Reverse	GCCCCACTTCCGGTCATCATCGTA	
E-cadherin	Forward	TATCGGATTTGGAGGGACACTG	151bp
	Reverse	GTGAACCATCATCTGTGGCGAT	
IL-6	Forward	TCCAGTTGCCTTCTTGGGAC	140bp
	Reverse	GTGTAATTAAGCCTCCGACTTG	
IL-10	Forward	GACTTTAAGGGTTACCTGGGTTG	112bp
	Reverse	TCACATGCGCCTTGATGTCTG	
TNF-a	Forward	CACGTCGTAGCAAACCACCAAGTGGA	140bp
	Reverse	TGGGAGTAGACAAGGTACAACCC	
β-Actin	Forward	CATGTACGTTGCTATCCAGGC	250bp
	Reverse	CTCCTTAATGTCACGCACGAT	

Table S2. Mass spectrometry information of active components in urine.

No.	RT (min)	Ion mode	Mass Accuracy (ppm)	Assigned identity	Molecular formula	Theoretical extract mass (Da)	HRMS(m/z)	Product ions of ESI/MS2
U1	28.036	[M+H]+1	0.79	Rupestonic acid	C15 H20 O3	248.14144	249.14868	231.10，105.07，91.05，81.07
U2	32.138	[M+H]+1	0.43	Chrysosplenetin B	C19 H18 O8	374.10033	375.10748	359.07，342.07，317.07，299.05
U3	35.266	[M+H]+1	-0.38	Artemetin	C20 H20 O8	388.11567	389.12296	373.09
U4	24.104	[M-H]-1	1.48	Ambrosic acid	C15 H20 O4	264.13655	263.12927	219.14，201.13
U5	28.984	[M+H]+1	0.95	Irigenin	C18 H16 O8	360.08486	361.09183	328.06，301.15，168.01
U6	22.001	[M+H]+1	1.21	Luteolin 7-glucuronide*	C21 H18 O12	462.08039	463.08731	287.05
U7	33.035	[M+H]+1	0.21	Isoalantolactone	C15 H20 O2	232.14638	233.15366	215.14，187.15，145.10，131.09，119.09
U8	23.059	[M+H]+1	0.99	Apigenin 7-O-glucuronide	C21 H18 O11	446.08535	447.09241	271.06，153.02，119.05
U9	27.802	[M+H]+1	0.7	Nabumetone	C15 H16 O2	228.11519	229.12247	172.09，171.08，156.09，128.06
U10	41.707	[M+H]+1	-0.24	Artemisinic acid	C15 H22 O2	234.16192	235.16919	84.08
U11	24.786	[M+H]+1	-1.22	Acacetin	C16 H12 O5	284.06813	285.07529	153.04
U12	23.537	[M+H]+1	0.97	Arglabin	C15 H18 O3	246.12583	247.1331	185.13，151.08，109.06
U13	18.686	[M+H]+1	0.85	Alminoprofen	C13 H17 N O2	219.12612	220.13339	174.11，90.05，73.05
U14	22.699	[M+H]+1	-0.73	Kaempferol	C15 H10 O6	286.04753	287.05478	117.02，97.03
U15	33.039	[M+H]+1	-0.29	Curcumenol	C15 H22 O2	234.16191	235.16919	217.12，189.13，147.03，135.08，105.07
U16	22.677	[M+H]+1	-0.29	Linderane	C15 H16 O4	260.10478	261.11206	243.10，173.10，156.09
U17	22.476	[M+H]+1	0.08	Artemisinin	C15 H22 O5	282.14675	283.15402	268.98
U18	23.711	[M+H]+1	0.78	Fraxinellone	C14 H16 O3	232.11012	233.1174	129.07，117.07
U19	25.651	[M+H]+1	-1.92	6-O-Methylscutellarin	C22 H20 O12	476.09456	477.10157	301.07，286.05，258.05，229.05
U20	30.046	[M+H]+1	-0.54	Crocetin	C20 H24 O4	328.16728	329.17456	197.13，91.05
U21	22.622	[M-H]-1	-1.41	Dihydroartemisinin	C15 H24 O5	284.16197	283.1547	268.04，240.04
U22	28.008	[M-H]-1	-2.51	Hispidulin	C16 H12 O6	300.06263	299.05558	267.03

U23	18.378	[M+H]+1	-3.3	Jasminoside B	$C_{16}H_{26}O_{8}$	346.16162	347.16921	149.1，121.10，105.07
U24	23.42	[M-H]-1	-2.25	Scutellarin	$C_{21}H_{18}O_{12}$	462.07879	461.07204	286.04，285.04，113.02
U25	25.852	[M-H]-1	-1.21	Oroxylin A-7-O-β-D-glucuronide	$C_{22}H_{20}O_{11}$	460.10001	459.09297	283.15

*** The component designated by the reference substance.**
